# Progranulin deficiency exacerbates spinal cord injury by promoting neuroinflammation and cell apoptosis in mice

**DOI:** 10.1186/s12974-019-1630-1

**Published:** 2019-11-27

**Authors:** Chao Wang, Lu Zhang, Jean De La Croix Ndong, Aubryanna Hettinghouse, Guodong Sun, Changhong Chen, Chen Zhang, Ronghan Liu, Chuan-ju Liu

**Affiliations:** 1 Department of Orthopaedic Surgery, New York University School of Medicine, New York, NY 10003 USA; 2 Department of Spine Surgery, The Affiliated Hospital of Qingdao University, Qingdao, 266000 Shandong China; 3 Department of Cell Biology, New York University School of Medicine, New York, NY 10016 USA

**Keywords:** Progranulin, Inflammation, Apoptosis, Spinal cord injury

## Abstract

**Purpose:**

Spinal cord injury (SCI) often results in significant and catastrophic dysfunction and disability and imposes a huge economic burden on society. This study aimed to determine whether progranulin (PGRN) plays a role in the progressive damage following SCI and evaluate the potential for development of a PGRN derivative as a new therapeutic target in SCI.

**Methods:**

PGRN-deficient (*Gr*^*−/−*^) and wild-type (WT) littermate mice were subjected to SCI using a weight-drop technique. Local PGRN expression following injury was evaluated by Western blotting and immunofluorescence. Basso Mouse Scale (BMS), inclined grid walking test, and inclined plane test were conducted at indicated time points to assess neurological recovery. Inflammation and apoptosis were examined by histology (Hematoxylin and Eosin (H&E) staining and Nissl staining, TUNEL assays, and immunofluorescence), Western blotting (from whole tissue protein for iNOS/p-p65/Bax/Bcl-2), and ex vivo ELISA (for TNFα/IL-1β/IL-6/IL-10). To identify the prophylactic and therapeutic potential of targeting PGRN, a PGRN derived small protein, Atsttrin, was conjugated to PLGA-PEG-PLGA thermosensitive hydrogel and injected into intrathecal space prior to SCI. BMS was recorded for neurological recovery and Western blotting was applied to detect the inflammatory and apoptotic proteins.

**Results:**

After SCI, PGRN was highly expressed in activated macrophage/microglia and peaked at day 7 post-injury. *Grn*^*−/−*^ mice showed a delayed neurological recovery after SCI at day 21, 28, 35, and 42 post-injury relative to WT controls. Histology, TUNEL assay, immunofluorescence, Western blotting, and ELISA all indicated that *Grn*^*−/−*^ mice manifested uncontrolled and expanded inflammation and apoptosis. Administration of control-released Atsttrin could improve the neurological recovery and the pro-inflammatory/pro-apoptotic effect of PGRN deficiency.

**Conclusion:**

PGRN deficiency exacerbates SCI by promoting neuroinflammation and cellular apoptosis, which can be alleviated by Atsttrin. Collectively, our data provide novel evidence of using PGRN derivatives as a promising therapeutic approach to improve the functional recovery for patients with spinal cord injury.

## Introduction

Spinal cord injury (SCI) is the most serious complication of spine trauma with potential to result in severe neurological dysfunctions [1]. Epidemiological data has indicated a population of approximately 282,000 persons affected by SCI in the USA in 2016, while the number of new cases was as high as 17,000 per year [2]. SCI not only brings catastrophic physical and psychological trauma to patients, but also incurs a huge economic burden to the society due to prolonged hospital admissions, poor rehabilitation outcome, and excessive nursing dependence [3]. The annual expenses of a high tetraplegia (C1-4) were estimated to be more than 1 million dollars in the first year, and as high as $185,111 for each subsequent year [2]. Evaluations of several pharmacological therapies aimed to improve SCI recovery have been initiated and are in various stages of clinical trials [4]. Nevertheless, the effectiveness and the safety of these therapies remain to be fully evaluated. The utility of the only neuroprotective drug which has been widely used in clinic for SCI, methylprednisolone, has been increasingly challenged for its controversial benefits, narrow treatment window, and side effects [5].

Progranulin (PGRN) is a 593 amino acid secreted glycoprotein that is widely expressed in many cell types including neurons, leukocytes, and chondrocytes [6]. As a pleiotropic growth factor-like protein, PGRN is involved in various physiological and pathological progresses, including embryogenesis, wound healing, host defense, tumorigenesis, and cartilage degeneration [7–9]. Accordingly, PGRN has been revealed as a potent therapeutic target in many disease models such as neurological diseases, inflammatory and autoimmune diseases, cancer, tissue repair, and lysosomal storage diseases [10–12]. PGRN has been shown to inhibit the inflammatory response in chronic inflammatory conditions such as rheumatoid arthritis [13], osteoarthritis [14], and inflammatory bowel disease [15], as well as acute inflammation such as acute lung injury [16], septic shock [17], and acute brain injury [18]. Our previous data has established that PGRN, as well as its engineered derivative, Atsttrin, directly binds to TNFR1 and TNFR2 to exert an anti-inflammatory effect in a murine model of rheumatoid arthritis [19]. In models of traumatic brain injury, PGRN knockout can aggravate neuroinflammatory response, axonal injury, and astrogliosis [20]. In contrast with what has been elucidated in brain injury models, the role of PGRN in SCI and the mechanism are quite obscure and remain to be illuminated. Naphade et al. previously demonstrated that PGRN was dramatically induced after SCI, primarily sourced from activated macrophage and microglia following injury; however, the significance of this phenomenon was not determined and has not received further investigation [21]. Herein, we conducted a series of experiments, to unlock the functional effect of PGRN deficiency, as well as the therapeutic effect of its derivative, Atsttrin, on SCI.

## Materials and methods

### Animals

PGRN-deficient female C57BL/6 mice (*Grn*^*−/−*^) and age/sex-matched littermate wild-type (WT) mice were used in this study. All mice were 20 to 25 g and 10 to 12 weeks old at the time of the experiment initiation. All animal studies were implemented in accordance with institutional regulations and approved by the Institutional Animal Care and Use Committee (IACUC) of New York University under study identification number IA15-01372. The mice were housed under standard conditions (22 ± 1 °C, 60 ± 5% relative humidity, 12-hour light/dark cycle, ad libitum access to food and water).

### Reagents

This study utilized a commercialized thermosensitive hydrogel (AK097, PolySciTech, Lafayette, USA) composed of PLGA-PEG-PLGA copolymers (LA:GA = 15:1 by weight) and previously proven to be an effective controlled-release carrier [22]. Hydrogel was dissolved at a 1:4 (v/v) ratio in PBS or in PBS plus Atsttrin. Final solutions were prepared as hydrogel (20%) with PBS or hydrogel (20%) with Atsttrin (0.64 μg/μl), respectively. Hydrogel preparations were carried out at 4 °C prior to use.

### Contusive spinal cord injury model and intrathecal hydrogel delivery

Mice were anesthetized with ketamine (50 mg/kg) and xylazine (3 mg/kg) via intraperitoneal injection. After careful dissection of skin, fascia, and paravertebral muscles, a T10 laminectomy was performed with stereotactic fixation of the spine. A total of 2.5 μl hydrogel/PBS or hydrogel/Atsttrin (a total of 1.6 μg) was delivered via intrathecal injection using a 33 G neuro-syringe (65460-06, Hamilton, Reno, USA). The beveled needle point of the syringe was dorsally punctured into intrathecal space at 45°, after which it was rotated ventrally towards the spinal cord and the hydrogel mixture was then injected into surface of the spinal cord. The mice were placed under a heating lamp for 2 min to accelerate coagulation of the hydrogel. Subsequently, a modified weight-drop Allen model was established as previously described [23]. For the contusive SCI model, an impactor (3 g weight, 1.5-mm diameter) was dropped from a height of 25 mm to the spinal cord to generate an immediate and moderate contusive injury. For the sham group, laminectomy was performed without contusive injury. The muscles and skin were aseptically sutured by layers. Sterile saline (100 μl/20 g body weight) and opioid analgesic, buprenorphine (50 μg/kg/day) were administered intraperitoneally immediately following skin closure and every 24 h for four consecutive days. Meanwhile, mice were intensively monitored twice a day to assess post-operative pain, weight loss, dehydration, wound healing, and infection. Mice had ad libitum access to water and standard rodent diet. Abundant soft pads were provided to prevent friction of the abdomen and genitals against the caging. Bladder massage was manually carried out twice a day to prevent urological infection until autonomic urination. No severe or unexpected complications occurred during the experiments.

### Neurological recovery assessment

Three methods, including Basso Mouse Scale, inclined grid walking test, and inclined plane test, were applied to comprehensively evaluate neurological recovery after SCI. After preliminary trial and power analysis, a minimum of 6 mice per group was adopted for the assessments.

#### Basso Mouse Scale

The motor function recovery of the hind limbs was assessed by Basso Mouse Scale (BMS) [24]. Briefly, two independent raters were trained for the BMS recording and blind to the grouping of the mice for the entire experiment. Mice were evaluated using the BMS pre-operatively and on post-operative days 1, 7, and then weekly until day 42. Each mouse was scored during free ambulation in an open field for 4 min using a 0 to 9 point rating system in accordance with standards in the scale.

#### Inclined grid walking

This trial was modified from the method described previously [25]. The mouse was placed at the bottom of a grid box (2 cm squares and 30 cm long) adjusted at a 45° slope. The number of hind falling (errors) from the grid was counted for the duration of the climb from the bottom to the top. All the mice were pre-trained for 3 consecutive days prior to surgery. Each trial was repeated three times and the average was recorded. Considering the healing of soft tissue and recovery of hind climbing, the recording time points were set at 21, 28, 35, and 42 days post-operatively.

#### Inclined plane test

For the inclined plane test, the mouse was placed on a plane equipped with an indicator for the degree of the plane’s incline [26]. The plane was then inclined at a rate of 2° per second, and the degree at which the mouse fell from the plane was recorded as the falling degree. Each trial was repeated three times and the average was recorded. As in the inclined grid walking test, the recording time points were 21, 28, 35, and 42 days post-operatively.

### Western blotting

The mouse was euthanized at indicated days after surgery. For time-dependent expression of PGRN after injury, mice were euthanized at 1, 3, 5, 7, and 14 dpi (*n* = 3 per time point), while inflammatory/apoptotic proteins were detected at 7 dpi (*n* = 3 per group). The injured spinal cord (0.5-cm long) was excised, snap frozen in liquid nitrogen, and transferred to − 80 °C until use. The tissue was grinded in liquid nitrogen and homogenized in RIPA lysis buffer containing 1% Protease/Phosphatase Inhibitor Cocktail (5872, Cell Signaling Technology, Danvers, USA) for 30 min at 4 °C. After centrifugation at 4 °C and 18,000×*g* for 20 min, the supernatant was collected and the concentration of total protein was quantified by using a BCA Protein Assay Kit (23227, Thermo Scientific, Waltham, USA). A total of 60 μg protein per lane were loaded to an 8% or 12% SDS-PAGE in accordance with appropriate ranges of molecular weight detection. After electrophoresis, the protein was transferred to a nitrocellulose (NC) membrane, followed by 1 h non-specific blocking with 5% non-fat milk in TBS-Tween 20 (0.1%) buffer. The membrane was then incubated with the indicated primary antibody at 4 °C overnight, and subsequently incubated with corresponding HRP-conjugated secondary antibody for 1 h at room temperature. The membrane was coated evenly with substrate solution (32106, Thermo Scientific, Waltham, USA) and visualized by using an enhanced chemiluminescence system (Amersham Life Science, Arlington Heights, USA). ImageJ software (NIH, New York, USA) was used for quantification.

The primary antibodies used for Western blotting included rabbit anti-GAPDH ( 1:1000, 2118, Cell Signaling Technology), rabbit anti-Phospho-NF-κB p65 (Ser536) (1:1000, 3033, Cell Signaling Technology), rabbit anti-NF-κB p65 (1:1000, 4764, Cell Signaling Technology), rabbit anti-Bax (1:1000, 5023, Cell Signaling Technology), mouse anti-Bcl-2 (1:200, sc-7382, Santa Cruz Biotechnology, Dallas, TX, USA), sheep anti-PGRN (1:2000, AF2557, R&D Systems, Minneapolis, MN, USA), and rabbit anti-iNOS (1:400, PA1-036, Invitrogen, Carlsbad, CA, USA).

### Ex vivo ELISA

Since the peripheral blood serum cannot precisely reflect the inflammation status of the spinal cord, and cerebrospinal fluid was technically difficult to acquire from the mice, an ex vivo assay was performed to detect the release of inflammatory cytokines after SCI. On day 7 post-injury, mice from WT-sham, WT-SCI, *Grn*^*−/−*^-sham and *Grn*^*−/−*^-SCI groups (*n* = 5 for each group) were anesthetized and the injured spinal cords were exposed aseptically. The area of spinal cord encompassing 5 mm from epicenter of the injury in each direction was excised promptly and transferred to DMEM (200 μl/10-mg spinal cord) without FBS in a 48-well plate. After incubation at 37 °C in a humidified incubator for 24 h, the supernatants were collected for ELISA assays. Inflammatory cytokines, including TNFα, IL-1β, IL-6, and IL-10, were detected by using mouse ELISA kits (Invitrogen, Carlsbad, USA) according to the manufacturer’s protocol.

### Immunofluorescence

The frozen sections (*n* = 3 per group) were first permeabilized using 0.1% Triton X-100 for 10 min followed by blocking with 5% donkey serum in PBS for 30 min. The sections were then incubated with primary antibodies overnight at 4 °C, subsequently rinsed with PBS, and incubated with secondary antibodies at RT for 1 h. After rinsing with PBS for 3 times, the slides were mounted using Vectashield mounting medium with DAPI (H-1200, Vector Laboratories, Burlingame, CA, USA). The slides were subsequently imaged under immunofluorescence microscope (Axio Scope.A1, Zeiss, Oberkochen, Germany).

The primary antibodies used in this study included mouse anti-NeuN (1:400, ab104224, Abcam, Cambridge, England), mouse anti-GFAP (1:100, sc-33673, Santa Cruz Biotechnology), rat anti-CD68 (1:200, MCA1957, Bio-Rad Laboratories, Hercules, USA), sheep anti-PGRN (1:200, AF2557, R&D Systems, Minneapolis, USA), and rabbit anti-iNOS (1:200, PA1-036, Invitrogen, Carlsbad, USA). The secondary antibodies used in this study included donkey anti-mouse Alexa Fluor 488 (1:200, A-11017, Invitrogen, Carlsbad, USA), donkey anti-rat Alexa Fluor 488 (1:200, A-21208, Invitrogen, Carlsbad, USA), donkey anti-rabbit Cy3 (1:200, AP182C, Sigma-Aldrich, Darmstadt, Germany), and donkey anti-sheep Alexa Fluor 647 (1:200, A-21448, Invitrogen, Carlsbad, USA)

### Section preparation

At day 7 after injury, the mice (*n* = 3 per group) were anesthetized and sacrificed by intracardial injection with 0.9% saline, followed by 4% PFA in PBS. The spinal cords (1 cm in each direction from the epicenter) were harvested and immediately fixed overnight in 4% PFA at 4 °C. The tissues were then immersed in 30% sucrose-PFA solution until they sunk to the bottom. The tissues were embedded in cryogel and snap frozen in liquid nitrogen. A cryostat (CM3050S, Leica, Wetzlar, Germany) was used to obtain12-μm thick transverse or longitudinal slices, which were stored at − 80 °C until use.

### TUNEL assay

At day 7 post-injury, the frozen transverse sections from both WT and *Grn*^*−∕−*^ mice (*n* = 3 respectively) were acquired. Sections from both 1 mm caudal and rostral from epicenter were subjected to the terminal deoxynucleotidyl transferase-mediated deoxyuridine triphosphate nick end labeling (TUNEL) assay with detection by the DeadEnd Colorimetric TUNEL system (Promega, Madison, USA) according to the manufacturer’s instructions. Methyl green (198080, Sigma-Aldrich) was used for counter staining. Images of the apoptotic cells were visualized by using a light microscope (Axio Scope.A1, Zeiss, Oberkochen, Germany). Total number of TUNEL-positive cells at the ventral horns was counted from each slide and the averages were recorded to quantitatively examine the numbers of apoptotic cells.

### Nissl staining

The slices from both WT and *Grn*^*−∕−*^ mice (*n* = 3 per group) at day 7 after SCI were removed from the freezer and kept at room temperature for 60 min. After rinse in ddH_2_O, the slides were immersed in cresyl violet solution (0.1% cresyl violet acetate and 0.25% glacial acetic acid in ddH_2_O) for 20 min. The slides were sequentially dipped in ddH_2_O, gradient ethanol (90%, 95%, and 100%), and xylene for dehydration. The slides were then coversliped with mounting medium and scanned using a light microscope (Axio Scope.A1, Zeiss, Oberkochen, Germany).

### Hematoxylin and Eosin staining

At day 7 post-injury, the frozen longitudinal sections from both WT and *Grn*^*−∕−*^ mice (*n* = 3 respectively) were acquired. H&E staining was performed in accordance with a well-established protocol [27]. After curing of the mounting medium, the sections were examined under a light microscope (Axio Scope.A1, Zeiss, Oberkochen, Germany) and the lesion areas were measured using ImageJ (NIH, New York, USA) software. The relative lesion area was defined as inflammatory area divided by area of the whole longitudinal spinal cord 2 mm from the epicenter of the injury.

### Statistical analysis

Data were presented as mean ± standard error of the mean (SEM) from three independent experiments. Statistical significance was examined by using Student’s *t* test when there were two compared groups. When more than two groups were compared, statistical evaluation of the data was performed by using one-way analysis of variance (ANOVA) and Turkey’s post hoc test. *p* values < 0.05 were considered to be statistically significant.

## Results

### PGRN is differentially upregulated in the course of SCI

**Fig. 1:**
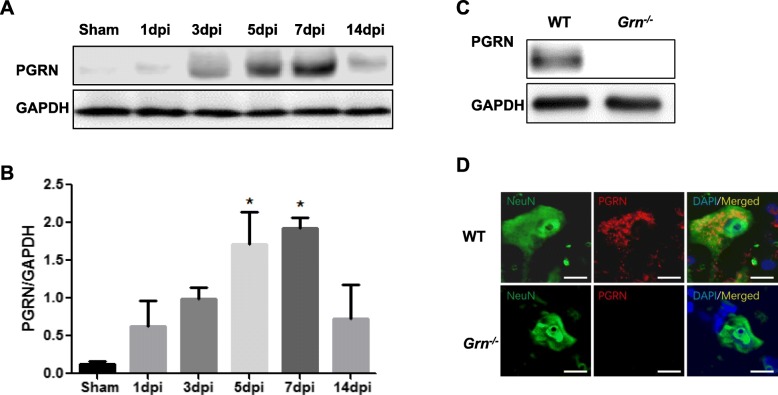
PGRN expression is enhanced in SCI. **a**, **b** Representative Western blotting of PGRN at different time points after SCI (**a**); Quantification of Western Blots with Image J (*n* = 3 per group) (**b**). One-way ANOVA and Turkey’s post hoc test were used. *p < 0.05, **p < 0.01 compared with sham group. **c**, **d** The specificity of the PGRN antibodies was confirmed by Western blotting (**c**) and immunofluorescence (**d**) in *Grn*^*−∕−*^ mice at 7 days after SCI

Compared with sham group (0.12 ± 0.05), the protein level of PGRN after SCI increased dramatically (*n* = 3; 0.63 ± 0.33, 0.99 ± 0.16, 1.71 ± 0.42, 1.93 ± 0.14, and 0.73 ± 0.45, for 1, 3, 5, 7, and 14 days post-injury (dpi), respectively). Statistical analysis showed that the difference became significant at 5 and 7 dpi (*p* < 0.05 and *p* < 0.01 respectively, vs sham group), with the peak at day 7 post-injury (Fig. 1a and b). The specificity of the PGRN antibodies is demonstrated by Western blotting (Fig. 1c) and immunofluorescence (Fig. 1d) of tissues harvested from WT and *Grn*^*−∕−*^ mice.

**Fig. 2:**
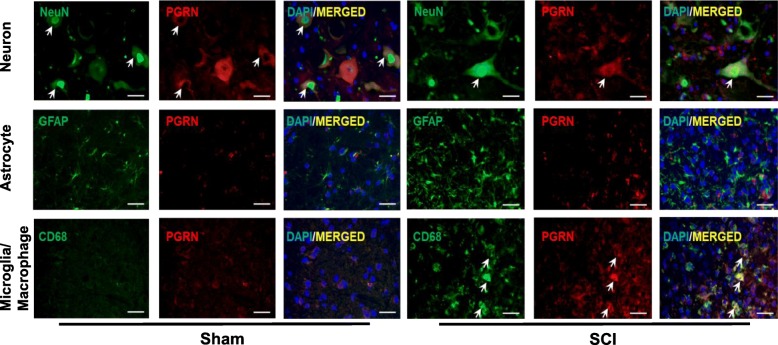
PGRN is mainly localized in neuron and activated macrophage/microglia after SCI. Immunofluorescence double staining of spinal tissues from sham and SCI groups with specific antibodies against PGRN (*red*) or cell markers (*green*): NeuN (neuron), GFAP (astrocyte), and CD68 (macrophage/microglia). Nuclei were stained by DAPI (*blue*), and *yellow* indicates merged image. Bar = 20 μm

We further explored the specific cell types that contributed to elevated PGRN level, and immunofluorescence was performed for both sham and SCI groups (Fig. 2). In the sham group, which mimicked the physiological condition of spinal cord, PGRN co-localization with CD68+ macrophage/microglia was hardly detectable and neuron was the main resource of PGRN. After SCI, PGRN co-localized with both NeuN and CD68, but not astrocyte marker GFAP, which indicated that neuron and activated macrophage/microglia were the predominant cell types that expressed PGRN after SCI.

### PGRN deficiency impairs neurological recovery, enlarges inflammatory area, and suppresses motor neuron survival after SCI

**Fig. 3:**
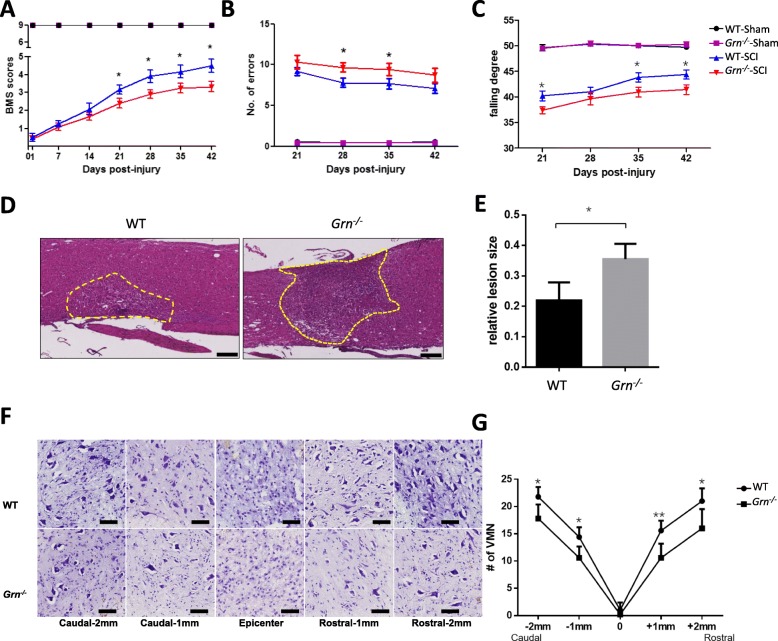
PGRN deficiency impairs neurological recovery, enlarges inflammatory area, and suppresses motor neuron survival after SCI**.** Assessments of BMS (**a**), inclined grid walking test (**b**), and inclined plane test (**c**) were used to evaluate functional recovery after sham or SCI between WT and *Grn*^*−∕−*^ groups. *n* = 6 for each group. **d** H&E staining from longitudinal sections showed the inflammatory area between WT and *Grn*^*−∕−*^ groups. Bar = 200 μm. **e** Quantification of (**d**). *n* = 3 per group. **f** Nissl staining from transverse sections showed typical views of ventral motor neurons (VMN) between WT and *Grn*^*−∕−*^ groups at different distances from the epicenter of injury. Bar = 50 μm. **g** Quantification of (**f**). *n* = 3 per group. *p < 0.05, ** *p* < 0.01 WT-SCI vs *Grn*^*−∕−*^-SCI groups

To assess the effect of PGRN deficiency on neurological recovery, three behavioral tests were implemented. Results showed that at day 1 post-injury, the BMS scores for WT-SCI and *Grn*^*−∕−*^-SCI groups lowered to 0.50 ± 0.22 and 0.42 ± 0.15, respectively, with *p* > 0.05, which indicated the successful establishment of the SCI model in both genotypes. At days 21, 28, 35, and 42 post-injury, the *Grn*^*−∕−*^-SCI group exhibited a significantly lower score (*n* = 6; 2.42 ± 0.27, 2.92 ± 0.24, 3.25 ± 0.28, and 3.33 ± 0.28) than the WT group (*n* = 6; 3.17 ± 0.25, 3.92 ± 0.35, 4.17 ± 0.36, and 4.50 ± 0.39, *p* < 0.05, Fig. 3a). For inclined grid walking test, the number of falling errors was significantly increased in *Grn*^*−∕−*^-SCI group at 28 dpi (*n* = 6; 9.67 ± 0.55 vs 7.78 ± 0.56, *p* < 0.05, Fig. 3b), while the falling degree of *Grn*^*−∕−*^-SCI group in the inclined plane test was significantly decreased at day 21, 35, and 42 post-injury (*n* = 6; 37.39 ± 0.67 vs 40.22 ± 0.96, 40.94 ± 0.96 vs 43.83 ± 0.91, 41.44 ± 0.95 vs 44.39 ± 0.89, *p* < 0.05, Fig. 3c).

H&E staining of the longitudinal sections showed an expanded inflammation area in the *Grn*^*−∕−*^-SCI group (*n* = 3; 0.22 ± 0.03 vs 0.35 ± 0.03, *p* < 0.05, Fig. 3d and e). Nissl staining for ventral motor neurons proved that, except in the epicenter area, the *Grn*^*−/−*^-SCI group showed more neuron loss at indicated directions and distances from the injury site (*n* = 3; caudal-2 mm 17.80 ± 1.16 vs 21.80 ± 0.80, *p* < 0.05; caudal-1 mm 10.60 ± 0.93 vs 14.40 ± 0.81, *p* < 0.05; rostral-1 mm 10.60 ± 1.17 vs 15.60 ± 0.81, *p* < 0.01; and rostral-2 mm 16.00 ± 1.58 vs 21.00 ± 1.05, *p* < 0.05, respectively, Fig. 3f and g).

### PGRN deficiency exacerbates inflammatory response and apoptosis after SCI

**Fig. 4:**
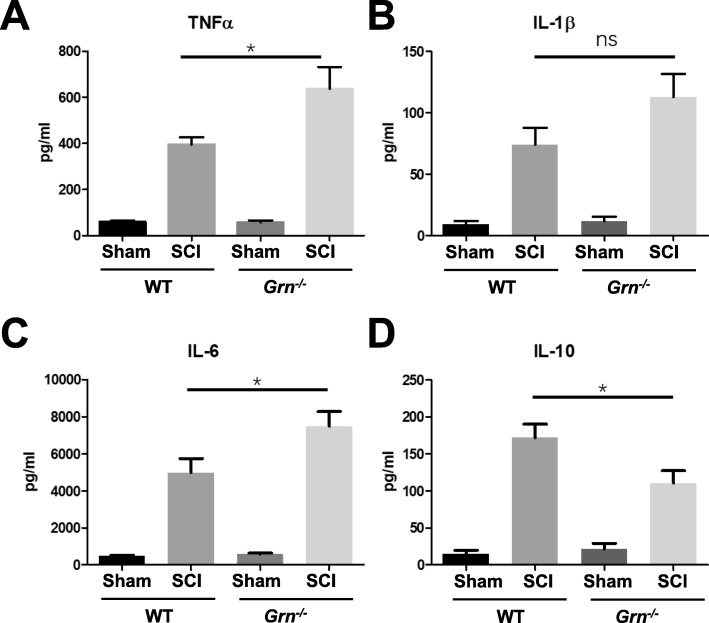
PGRN deficiency promoted neuroinflammation by ex vivo ELISA test. Supernatants of ex vivo tissue cultures were measured by ELISA for TNFα (**a**), IL-1β (**b**), IL-6 (**c**), and IL-10 (**d**). *n* = 5 per group. One-way ANOVA and Turkey’s post hoc test were used. *p < 0.05 WT-SCI vs *Grn*^*−∕−*^-SCI groups. ns, not significant

To compare the inflammatory response between groups, we firstly examined the release of pro- and anti-inflammatory cytokines. Considering that the levels of cytokines in peripheral blood might not accurately reflect the status of the CNS due to the blood-spinal cord barrier, compounded by the technical difficulty in collection of sufficient cerebrospinal fluid, we used segmental spinal cord ex vivo culture and supernatant cytokine detection by ELISA to assess inflammatory response. As shown in Fig. 4, the levels of pro-inflammatory cytokines TNFα (Fig. 4a) and IL-6 (Fig. 4c) were significantly higher in the *Grn*^*−∕−*^-SCI group than in the WT-SCI group (*n* = 5; 637.2 ± 94.52 vs 394.4 ± 31.29, and 7490 ± 792.0 vs 4980 ± 753.4, respectively), though another pro-inflammatory cytokine, IL-1β (Fig. 4b), showed no statistical difference (113.0 ± 18.88 vs 74.08 ± 13.71). Anti-inflammatory cytokine IL-10 (Fig. 4d) manifested a significant decrease compared with WT group (110.5 ± 16.83 vs 172.1 ± 18.56).

**Fig. 5:**
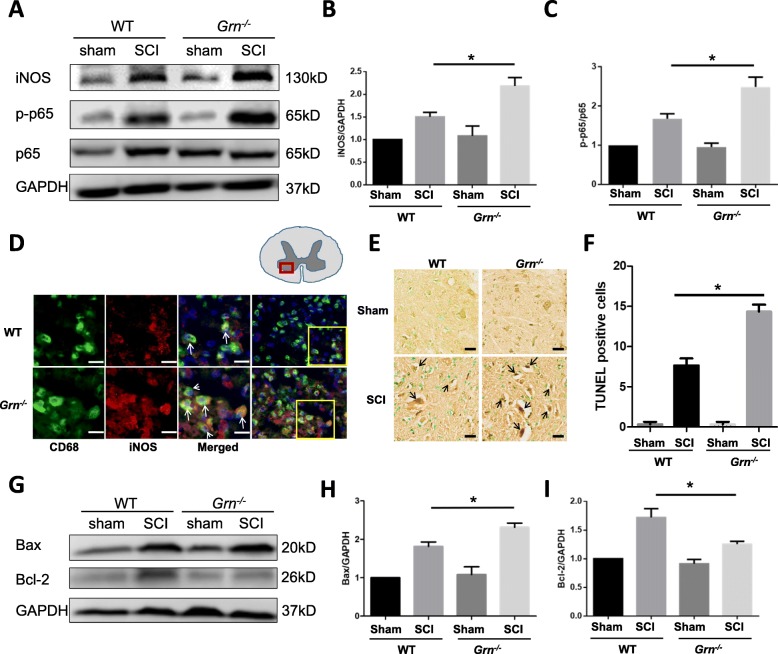
PGRN deficiency aggravates neuroinflammation and apoptosis after SCI. **a** At day 7 after SCI, total protein was extracted from injured spinal cord of both WT and PGRN-deficient mice, and iNOS, p-p65, p65, and GAPDH were detected by Western blotting. **b** Quantification of iNOS/GAPDH in (**a**). *n* = 3 per group. **c** Quantification of p-p65/p65 in (**a**). *n* = 3 per group. **d** Immunofluorescence of iNOS and CD68 in both WT and PGRN-deficient mice at day 7 after SCI. Images were taken at the anterior horn of gray matter. Arrows indicate co-localization of iNOS and CD68. **e** At day 7 after SCI, total protein was extracted from injured spinal cord of both WT and PGRN-deficient mice, and Bax, Bcl-2, and GAPDH were detected by Western blotting. **f** Quantification of Bax/GAPDH in (**e**). *n* = 3 per group. **g** Quantification of Bcl-2/GAPDH in (**e**). *n* = 3 per group. **h** At day 7 after SCI, apoptotic neurons in the ventral horn were detected by TUNEL assays. **i** Quantification of apoptotic neuron numbers in (**h**). *n* = 3 for each group. *p < 0.05, ** *p* < 0.01 WT-SCI vs *Grn*^*−∕−*^-SCI groups. ns, not significant

Further, we examined one of the most important inflammatory products, iNOS, and the key protein in NF-κB pathway, phosphorylated p65. Compared with the WT mice, PGRN deficiency mice had elevated level of both iNOS (*n* = 3; 2.18 ± 0.19 vs 1.50 ± 0.10, *p* < 0.05) and p-p65 (*n* = 3; 2.49 ± 0.25 vs 1.67 ± 0.13, *p* < 0.05, Fig. 5a–c) following injury. Immunofluorescence of iNOS and CD68 showed that iNOS co-localized with activated macrophage/microglia (Fig. 5d arrow). TUNEL assay, which is widely used for detection of apoptotic cells, showed that PGRN-deficient mice had more apoptotic cells at ventral horn of the spinal cord (*n* = 3; 14.33 ± 0.88 vs 7.67 ± 0.88, *p* < 0.01, Fig. 5e and f). To verify this effect, another two mitochondrial apoptosis-associated markers, Bax (pro-apoptotic protein) and Bcl-2 (anti-apoptotic protein), were examined by Western blotting. Quantification showed that *Grn*^*−∕−*^ mice had increased Bax (*n* = 3; 2.31 ± 0.11 vs 1.82 ± 0.11, *p* < 0.05) and decreased Bcl-2 (*n* = 3; 1.25 ± 0.05 vs 1.72 ± 0.15, *p* < 0.05, Fig. 5g–i), which were in accordance with TUNEL assay.

### Local delivery of Atsttrin ameliorates neuroinflammation and apoptosis in PGRN-deficient mice after SCI

**Fig. 6:**
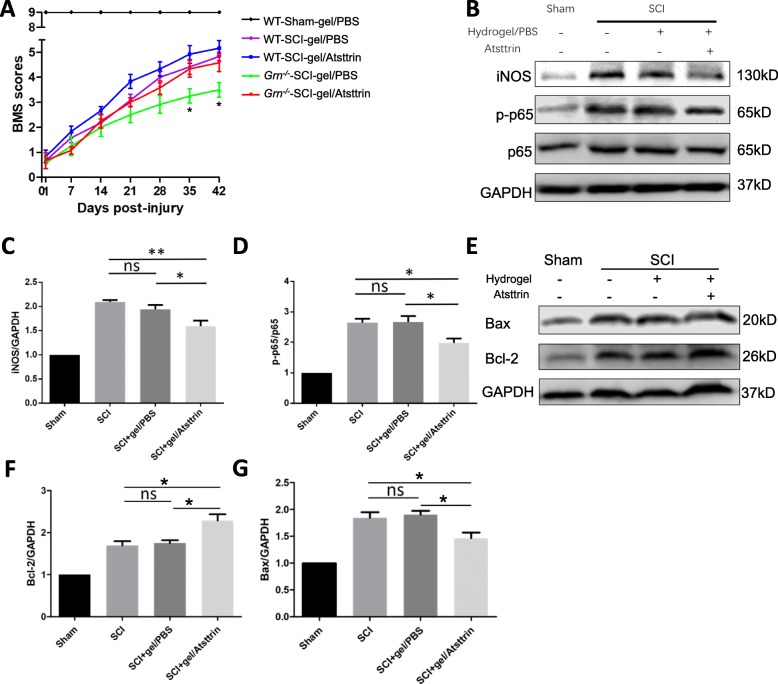
Local delivery of Atsttrin improves neuroinflammation and apoptosis in PGRN-deficient mice after SCI. **a** BMS was used to evaluate the therapeutic effect of hydrogel/Atsttrin in both WT and PGRN-deficient mice. Asterisk indicates *p* < 0.05 between *Grn*^*−∕−*^-SCI-gel/PBS and *Grn*^*−∕−*^-SCI-gel/Atsttrin groups. **b** At day 7 after SCI, total protein was extracted from injured spinal cord of PGRN-deficient mice and iNOS, p-p65, p65, and GAPDH were detected by Western blotting. Quantification of iNOS/GAPDH (**c**) and p-p65/p65 (**d**) in (**b**). *n* = 3 for each group. **e** At day 7 after SCI, total protein was extracted from injured spinal cords of PGRN-deficient mice and Bax, Bcl-2, and GAPDH were detected by Western blotting. Quantification of Bax/GAPDH (**f**) and Bcl-2/GAPDH (**g**) in (**e**). *n* = 3 for each group. *p < 0.05, **p < 0.01 for indicated groups, ns, not significant

To assess the therapeutic effect of Atsttrin, we designed a control released system by using a commercialized hydrogel as the carrier and injected the hydrogel/PBS or hydrogel/Atsttrin to the surface of the spinal cord prior to SCI. BMS scoring showed that *Grn*^*−∕−*^ mice receiving Atsttrin injection exhibit functional improvements at 35 dpi (*n* = 6; 4.33 ± 0.33 vs 3.25 ± 0.28, *p* < 0.05) and 42 dpi (*n* = 6; 4.58 ± 0.35 vs 3.50 ± 0.29, *p* < 0.05), while there was no statistical difference for WT mice with or without Atsttrin (Fig. 6a). To clarify the effect of Atsttrin in PGRN-deficient mice, we detected representative inflammatory proteins (iNOS and p-p65, Fig. 6b–d) and apoptotic proteins (Bax/Bcl-2, Fig. 6e–g). While there was no difference between SCI mice and SCI+hydrogel/PBS mice, the SCI+hydrogel/Atsttrin group showed significant decrease for both iNOS (*n* = 3; SCI vs SCI+gel/PBS vs SCI+gel/Atsttrin, 2.01 ± 0.04 vs 1.94 ± 0.09 vs 1.59 ± 0.12) and p-p65 (*n* = 3; SCI vs SCI+gel/PBS vs SCI+gel/Atsttrin, 2.65 ± 0.13 vs 2.67 ± 0.20 vs 1.98 ± 0.15). Accordingly, administration of Atsttrin dramatically increased Bcl-2 (*n* = 3; SCI vs SCI+gel/PBS vs SCI+gel/Atsttrin, 1.69 ± 0.10 vs 1.75 ± 0.07 vs 2.29 ± 0.15) and decreased Bax (*n* = 3; SCI vs SCI+gel/PBS vs SCI+gel/Atsttrin, 2.01 ± 0.04 vs 1.94 ± 0.09 vs 1.59 ± 0.12) after SCI, suggesting an anti-apoptotic effect of Atsttrin.

## Discussion

In the present study, we investigated the effect of PGRN deficiency on SCI in a well-established murine model. Our data indicate that PGRN deficiency results in an exaggerated inflammatory response and enhanced neuronal cell death following injury, which contribute to impaired neurological recovery. Importantly, local administration of Atsttrin to *Grn*^−∕−^ mice can attenuate inflammation and neuronal death in our system, suggesting PGRN and its derivative as potential therapeutics for SCI.

PGRN is a widely expressed glycoprotein with pleiotropic functions and former studies have well discussed the anti-inflammation effect of PGRN in several inflammatory disease models [6, 28]. Heterozygous mutation of the GRN gene is the major cause of FTD-TDP, which is a subtype of frontotemporal dementia (FTD) characterized by ubiquitinated and fragmented TAR-DNA binding protein-43 (TDP-43) [29, 30]. Meanwhile, the polymorphism of *Grn* gene is associated with the late-onset Alzheimer’s disease [31] and exogenous addition of PGRN can protect against amyloid beta deposition and toxicity in an Alzheimer’s animal model [32, 33]. Besides neurodegenerative diseases, PGRN also acts as a potential therapeutic target for neurological injuries, such as subarachnoid hemorrhage (SAH) [34], acute ischemic stroke [35], and neural injury [20] following spinal contusion. Naphade et al*.* demonstrated that PGRN was mainly co-localized with myeloid cell markers CD11b and CD68 and dramatically upregulated after experimental spinal cord injury [21]. The induction of PGRN after spinal cord injury was further confirmed by microarray analysis [36]. Our results further confirmed that the expression level of PGRN protein is dramatically increased in neurons and activated macrophage/microglia after SCI, which may rely on a negative feedback mechanism. Significantly, we illustrate a protective benefit of PGRN by comparing WT and *Grn*^−∕−^ mice, and evaluate the novel therapeutic effect of Atsttrin in SCI based on data from *Grn*^−∕−^ mice. Three behavioral tests were employed in this study (BMS scores, inclined grid walking test, and inclined plane test) and the results show consistently impaired neurological recovery rate in *Grn*^−∕−^ mice after SCI relative to WT controls. Furthermore, intrathecal administration of Atsttrin could prevent traumatic injury-induced neurological deficits and improve post-injury neurological functions.

SCI consists of a two-step process including a primary immediate mechanical injury followed by an inflammatory process and apoptosis, which is characterized by activation of glial cells and infiltration of leukocytes that exacerbates tissue damage by releasing reactive oxygen species, pro-inflammatory cytokines/chemokines, proteases, and lysosome enzymes [37, 38]. In addition to the major pro-inflammatory transcription factor NF-κB-mediated neuroinflammatory responses, nitric oxide (NO), also play key roles in pathophysiology of SCI [39–41]. Based on our results, PGRN can act as a protective target by regulating the inflammatory response after SCI. On the one hand, PGRN deficiency aggravated the release of pro-inflammatory cytokines TNFα and IL-6 while the release of anti-inflammatory cytokine IL-10 was lessened in injured tissues. On the other hand, *Grn*^−∕−^ macrophage/microglia presented higher levels of iNOS and p-p65, suggesting an activating status of neural inflammatory response. As expected, mice treated with Atsttrin demonstrated markedly reduced inflammatory response. Collectively, our findings support the notion that PGRN is a key regulator of inflammation in SCI.

Atsttrin is the “minimal” engineered molecule that retains affinity to TNFR1/2 and could inhibit several downstream events of TNF/TNFR signaling [10, 19]. Atsttrin was more effective than rPGRN in delaying the onset of inflammation in two different mouse models of rheumatoid arthritis: collagen antibody–induced arthritis (CAIA) and collagen-induced arthritis (CIA) [19]. In the SCI model, *Grn*^−∕−^ mice treated with Atsttrin revealed prominently rescued neural function while wild-type mice treated with Atsttrin showed a higher, but not statistically significant BMS score. Given that the accumulative expression of endogenous PGRN after injury may counteract the therapeutic effect of Atsttrin, a higher administration of rPGRN/Atstrrin may be required for better treatment effect in wild-type mice. Based on these and previous results, we conclude that the anti-inflammation and anti-apoptosis effect of PGRN could, at least in part, depend on the activation of TNFR signal pathway; however, the contributions of TNFR1 and TNFR2 in the therapeutic effects of Atsttrin need to be further illustrated.

Inflammation raised by activation of macrophages and resident microglia is a key component in the progression of SCI. Bone marrow–derived macrophages (BMDMs) from PGRN-deficient mice produce more pro-inflammatory cytokines and less anti-inflammatory IL-10 than wild-type macrophages under bacterial endotoxin treatment in vitro [8]. Recent study has shown that PGRN is produced in CD68-positive microglia and suppresses excessive inflammatory responses related to activated microglia after traumatic brain injury (TBI) in mice [42]. In accordance with previous reports, we found that PGRN was co-localized with neuron as well as activated macrophage/microglia after SCI, attracting our attention to the importance of macrophage-derived PGRN in the pathology of SCI. Macrophages/microglia exist in two states: M1 phenotype that confers pro-inflammatory effects and M2 phenotype that confers anti-inflammatory effects [43]. Kigerl et al. have reported that the SCI site is comprised predominantly of M1 macrophages, with transient presence of M2 macrophages during the first 7 days of SCI [44]. Based on the published reports and our findings, we consider the possibility that PGRN or its derivatives could switch M1/M2 macrophage phenotypes after SCI in some ways, thus reinforcing the anti-inflammatory effect, which needs to be further investigated.

The damage of neurons and glial cells that are not effectively replaced after the injury is one of the main causes of disability after SCI. Cell death during secondary injury after SCI is caused partly by the activation of apoptotic mechanisms. In this study, we verified the anti-apoptotic effect of PGRN by examining the apoptosis-associated markers by TUNEL staining and Western blot analyzing for Bax/Bcl-2 protein. Previous study showed that PGRN could reduce neuronal apoptosis after subarachnoid hemorrhage by activation of Sortilin 1 signaling pathways [34, 45]. PGRN can reduce neuronal apoptosis by mitigating endoplasmic reticulum (ER) stress in reactive astrocytes therefore contributing to the alleviation of cerebral ischemic/reperfusion (I/R) injury. However, the underlying mechanism of PGRN on neuronal death in central nervous system (CNS) trauma, especially in SCI, remains largely unknown. Among post-traumatic secondary biochemical responses, signs of autophagy have also been detected. Autophagy is a lysosome-dependent essential cellular catabolic pathway and usually considered cytoprotective under most circumstances [46]. Inhibition of mTOR signaling using rapamycin during the acute phase of SCI reduces secondary damage at lesion sites and confers neuroprotective effects [47]. Besides, homozygous loss-of-function GRN mutation leads to a rare adult-onset form of neuronal ceroid lipofuscinosis (NCL). Recent evidences showed lysosomal dysfunctions in PGRN knockout microglia/macrophages suggesting PGRN may function in lysosomal homeostasis and autophagy [48, 49]. In all, exploration of the scope of PGRN in autophagy has great significance. We will perform further in vivo and in vitro experiments to better understand the relation of PGRN and autophagy in SCI pathology.

In summary, the present study demonstrates that PGRN deficiency exacerbates spinal cord injury through promoting neuroinflammation and cell apoptosis, and provides a rPGRN derivative, Atsttrin, as a potential therapeutic target in acute spinal cord injury.

## Conclusion

PGRN deficiency exacerbates SCI by promoting neuroinflammation and cellular apoptosis, which can be alleviated by Atsttrin. Collectively, our data provide novel evidence of using PGRN derivatives as a promising therapeutic approach to improve the functional recovery for patients with spinal cord injury.

## Data Availability

All data generated or analyzed during this study are included in this published article.

## Abbreviations

SCI: Spinal cord injury
PGRN: Progranulin
WT: Wild type
BMS: Basso Mouse Scale
IACUC: Institutional Animal Care and Use Committee
TUNEL: Terminal deoxynucleotidyl transferase-mediated deoxyuridine triphosphate nick end labeling
H&E: Hematoxylin and Eosin
SEM: Standard error of the mean
TBI: Traumatic brain injury
CNS: Central nervous system
NCL: Neuronal ceroid lipofuscinosis
dpi: Days post-injury
